# A case report: rapid progression of coronary atherosclerosis in a patient taking Targretin (Bexarotene)

**DOI:** 10.1186/s40959-020-00087-3

**Published:** 2020-12-14

**Authors:** Sean DeAngelo, Kailyn I. Mann, Muhammad Abdulbasit, Amy Ahnert, Deborah W. Sundlöf

**Affiliations:** 1 University of South Florida/Lehigh Valley Hospital, 1200 South Cedar Crest Blvd, Allentown, PA 18103 USA; 2 Lehigh Valley Health Network, Heart Institute, Allentown, PA USA; 3 Lehigh Valley Health Network - Muhlenberg Department of Cardiology, Bethlehem, PA USA; 4 Philadelphia College of Osteopathic Medicine, Philadelphia, PA USA

**Keywords:** Targretin (Bexarotene), Atherosclerosis, Cutaneous T-cell lymphoma, Atypical angina, Primary hypertriglyceridemia

## Abstract

Anti-neoplastic drugs have made major advancements in oncology, however they are not without cardiovascular consequences. We present a patient with cutaneous T-cell lymphoma receiving Targretin therapy who presented with accelerated atherosclerosis. His triglyceride level (TG) was greater than 1000 mg/dL, which rapidly improved with discontinuation of Targretin.

## Clinical history

Patient is a 56-year-old male who presented with atypical angina. He has a past medical history of cutaneous T-cell lymphoma (CTCL), type II diabetes, Tourette’s syndrome, mixed hyperlipidemia, former tobacco use disorder, and coronary artery disease status post drug eluding stent (DES) to the right coronary artery (RCA) in 2006. Family history is significant for premature coronary artery disease in his mother. The diagnosis of stage IV CTCL was confirmed by biopsy in 2009. He was subsequently started on narrow band UV-B phototherapy and interferon alpha. Therapy with Targretin was considered but at that time his TG were 458 mg/dL. He was initiated on fenofibrate 48 mg daily, Omega-3 acid ethyl esters 4 g daily and atorvastatin 20 mg daily. His TG level improved 290 mg/dL and 2 months later Targretin was initiated.

**Fig. 1:**
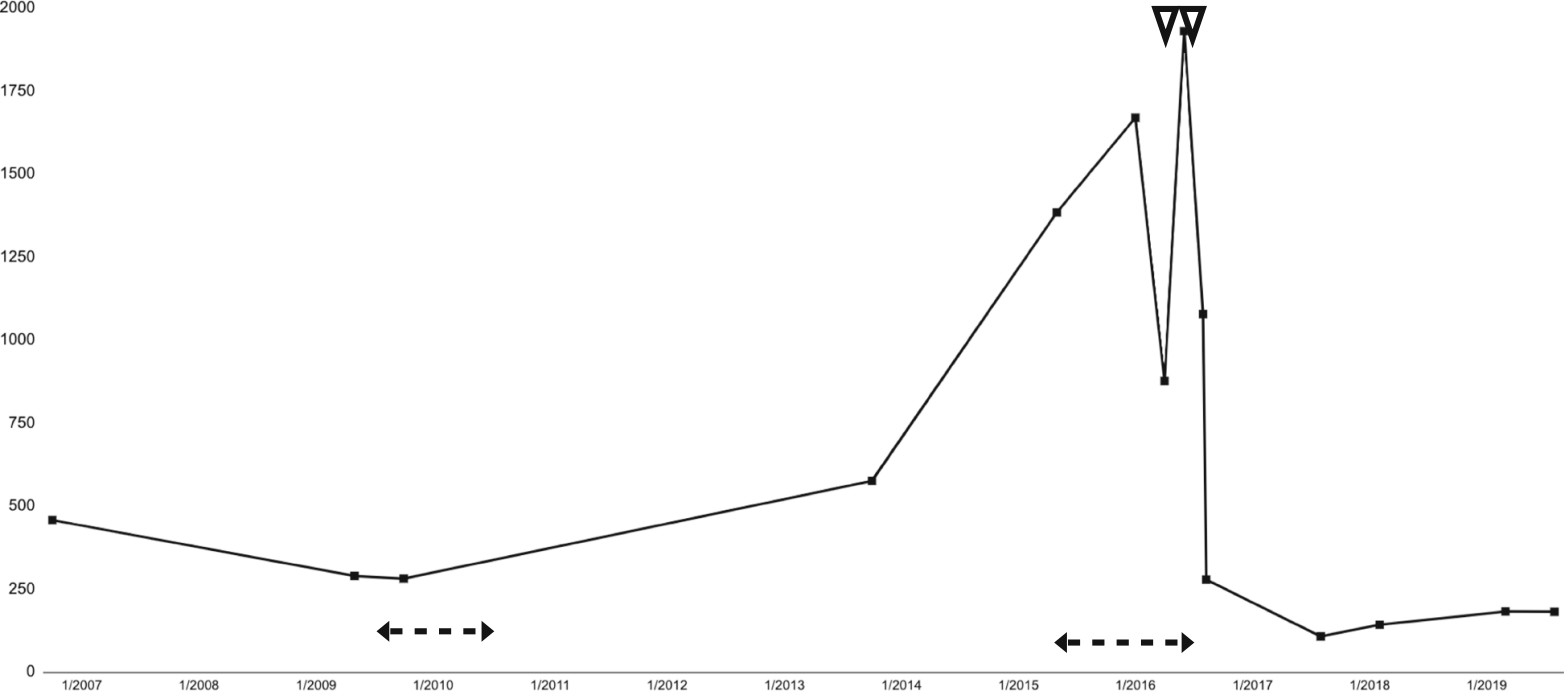
Comparison of a patient’s fasting triglyceride levels on Targretin vs off Targretin and identification of percutaneous coronary intervention. Filled squares (□) represent serum triglyceride levels. The dashed lines (|- - -|) represent the period of time the patient was on Targretin. The inverted arrows (▽) designate PCI with stent placement. The x-axis represents date (Month/Year) and the y-axis represents serum triglycerides (mg/dL)

The following year a PET/CT scan showed transformation of CTCL to Nodal Large Cell Lymphoma. Targretin was discontinued and a regimen of vincristine, doxorubicin, and cyclophosphamide was initiated. After 4 cycles, his chemotherapy regimen was advanced to rituximab, ifosfamide, carboplatin, and etoposide. Finally, a combination of methotrexate and cytarabine lead to remission in 2011. The following years, he had multiple relapses and remissions utilizing non-myeloablative stem cell transplant, six cycles of romidepsin, external beam radiation, and nitrogen mustard until systemic therapy with Targretin was restarted in 2015 at 300 mg daily (Fig. 1).

## Results

**Fig. 2:**
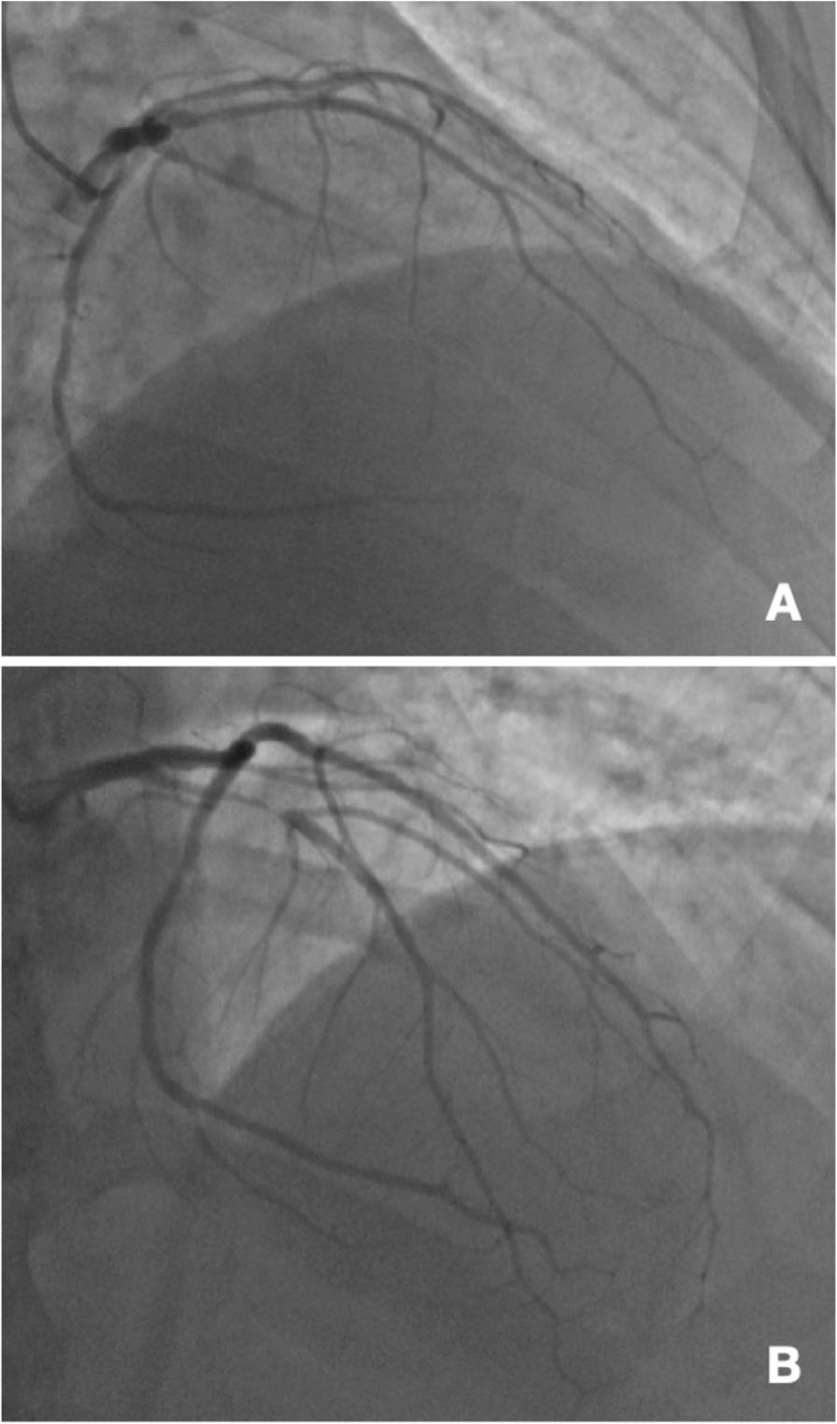
Comparison of PCI for a patient on Targretin therapy complicated by elevated triglycerides. **a** PCI required on May 31, 2016: the proximal segment of the RCA indicates prior stent placement in 2006 with 0% residual occlusion. The distal segment shows 50% occlusion. The LAD shows a 50% occlusion in the proximal segment. The Obtuse Marginal 1 segment is 95% occluded. A DES was placed resulting in 0% residual occlusion. The Obtuse Marginal 2 has 40% occlusion. **b** PCI required 8 weeks later on July 29, 2016: the LAD shows 95% occlusion in the proximal segment, in which a DES was placed; the result was 0% occlusion. The distal segment of the RCA indicates a reduction from 50 to 30%. The OM2 diameter was not measured in the second study

In 2016 the patient presented to the Emergency Department with atypical angina. The chest pain was ongoing for weeks and was described as a localized burning sensation without radiation. The pain occurred sporadically with both rest and exertion, lasting minutes and resolving spontaneously. Troponins were negative and a Dobutamine stress echocardiogram was negative for ischemia. He reported “heart burn” at peak Dobutamine infusion that resolved after drinking soda. He was seen in the cardiology office for follow up and reported persistent symptoms. Given his risk factors and continued chest pain syndrome, a cardiac catheterization was performed. He was found to have a 50% stenosis of his left anterior descending artery (LAD), 50% stenosis of his mid-RCA, patent prior proximal RCA stent, and a 95% stenosis of his obtuse marginal, which was treated with a DES. Anatomy and degrees of stenosis were visually estimated. No intravascular imaging techniques were utilized to provide further characterization (Fig. 2a). A lipid panel was checked at that time and showed a TG level of 877 mg/dL. His atorvastatin was increased to 40 mg and fenofibrate to 160 mg daily and he was maintained on omega-3 acid ethyl esters 4 g daily. He was also discharged on aspirin 81 mg daily and clopidogrel 75 mg daily. Beta-blocker therapy was deferred due to sinus bradycardia.

Two months later, the patient presented to the emergency department with recurrent anginal chest pain. It was characterized as a 6/10 in intensity, burning, sub-sternal chest pain with radiation to his left shoulder that came with walking up hill and resolved with rest. He underwent an exercise stress echocardiogram that was positive for LAD territory ischemia with a hypotensive blood pressure response. A repeat cardiac catheterization was performed and showed progression of the prior 50% LAD lesion to 95% for which a DES was placed. Similar to the previous catheterization procedure, the degree of stenosis was visually estimated and unaccompanied by intravascular visualization techniques (Fig. 2b). A repeat lipid panel was ordered and despite medical therapy with high dose atorvastatin and fenofibrate, his TG level exceeded 1000 mg/dL.

The patient was referred to a lipid specialist who performed lipid testing. Apolipoprotein-B and LDL levels were within goal ranges, at 116 g/L and 103 mg/dL, respectively, indicating this was primary hypertriglyceridemia. Targretin therapy was discontinued as it was thought to be a contributor to his rapidly progressive atherosclerosis. After discontinuing Targretin, his TG’s decreased to below 200 mg/dL.

His most recent lipid panel showed a TG of 108 mg/dL. He has not had any recurrent angina and CTCL is in remission without recurrent symptoms.

## Discussion

Hyperlipidemia is a known complication of individuals with CTCL on Targretin therapy. Fasting TG levels 2.5 times the upper limit of normal were noted in about 70% of patients on > 300 mg/m twice daily [1]. Current guidelines for safe prescribing of Targretin are based on a U.K. consensus statement. Initiation of Targretin requires TG levels below 150 mg/dL and fenofibrate 160-200 mg daily regardless of lipid status^2^. Screening for APOA5 and APOC3 status may potentially identify individuals at risk for poor TG response. APOA5 and APOC3 carriers are associated with significantly lower levels of TG compared to non-carriers in patients taking Targretin [3].

Maintenance of Targretin is based on dose-response. However, if TGs continually elevate, rosuvastatin, fenofibrate, omega-3 fatty acid and niacin combination is utilized to lower back to baseline. If TG levels are above 885 mg/dL, Targretin therapy should be discontinued [2]. A recent case report noted a return to baseline TGs as quick as 5 days after discontinuation of therapy [4].

If a patient has advanced refractory CTCL with limited treatment options, permissive elevation of TGs on Targretin should be discussed with the patient, including the risks [2]. Most notably, acute pancreatitis has been reported in patients taking Targretin with TG levels above 770 mg/dL [1]. Therefore, amylase, lipase and TG levels must be monitored closely in patients opting for permissive elevation of TGs. There are no current guidelines as to how often these levels should be checked in this patient population. Furthermore, there are no guidelines for the management of elevated TGs in prevention of coronary artery disease.

In our patient, multiple risk factors for cardiovascular disease were present on admission. He had a TIMI score of 2, ASCVD of 51.9% and a HEART score of 5 [5–7]. These calculators do not consider his severely elevated triglyceride levels (1930 mg/dL). Despite many years of debate, triglycerides are an important risk factor for cardiovascular disease, although it is unclear if TG’s are directly atherogenic [8]. Regardless of the calculated risks of future cardiac events, the patient’s intermediate pre-test probability for coronary artery disease was an indication for exercise testing [9]. Ensuing management with percutaneous intervention and discontinuation of Targretin allowed for successful resolution of the patients anginal symptoms and elevated serum TG in the 2 year follow up.

The rapid evolution of stenosis as described in the patient may suggest plaque destabilization in the LAD. Due to the lack of intravascular imaging techniques, the characterization of a pathophysiologic mechanism is limited. When considering Targretin therapy in a patient with a prior history of CAD and primary hypertriglyceridemia, a prescriber may regard the risk to outweigh the benefit.

## Conclusion

This is the first described case of accelerated coronary atherosclerosis on Targretin therapy requiring two primary percutaneous coronary interventions within 8 weeks. The TG levels normalized shortly after discontinuation of Targretin. Management focuses on optimizing patient risk factors for the development of cardiovascular disease.

## Data Availability

Not applicable.

## Abbreviations

CTCL: Cutaneous T-Cell Lymphoma
DES: Drug Eluding Stent
TG: Triglycerides
RCA: Right Coronary Artery
LAD: Left Anterior Descending
